# Sarcopenia: Marcadores Inflamatórios e Humorais em Pacientes Idosos com Insuficiência Cardíaca

**DOI:** 10.36660/abc.20220369

**Published:** 2023-07-27

**Authors:** Tamirys Delazeri Sangali, Gabriela Corrêa Souza, Édina Caroline Ternus Ribeiro, Ingrid Dalira Schweigert Perry

**Affiliations:** 1 Universidade Federal do Rio Grande do Sul Porto Alegre RS Brasil Universidade Federal do Rio Grande do Sul, Porto Alegre, RS – Brasil; 2 Hospital de Clínicas de Porto Alegre Porto Alegre RS Brasil Hospital de Clínicas de Porto Alegre, Porto Alegre, RS – Brasil

**Keywords:** Sarcopenia, Biomarcadores, Inflamação, Insuficiência Cardíaca

## Abstract

**Fundamento:**

Sarcopenia é altamente prevalente em pacientes com insuficiência cardíaca (IC), e o envolvimento de biomarcadores em sua fisiopatologia é sugerido, mas poucos estudos foram realizados em relação a pacientes sarcopênicos com IC.

**Objetivos:**

Avaliar a associação entre marcadores inflamatórios e humorais e sarcopenia, bem como o impacto da sarcopenia na qualidade de vida e na capacidade funcional em pacientes mais velhos com IC.

**Métodos:**

Neste estudo transversal, 90 pacientes ambulatoriais com IC, com idade ≥60 anos, foram avaliados quanto a sarcopenia (critérios diagnósticos EWGSOP2), inflamação (Proteína C reativa de alta sensibilidade [PCR-as], Interleucina-6 [IL-6], fator de necrose tumoral alfa [TNF-α]) e marcadores humorais (testosterona total e fator de crescimento semelhante à insulina tipo 1 [IGF-1]), atividade física (Questionário internacional de atividade física), qualidade de vida ( *Minnesota Living with Heart Failure Questionnaire* – Questionário Minnesota sobre conviver com a insuficiência cardíaca), e capacidade funcional (teste de caminhada de 6 minutos). O nível de significância estatística adotado foi p <0,05.

**Resultados:**

Os pacientes tinham uma média de idade de 69,4 ± 7,2 anos, 67,8% eram do sexo masculino, com fração de ejeção ventricular esquerda (FEVE) de 35,9 ± 11,9% e 22 (24,4%) eram sarcopênicos. Idade (73,1 ± 8,1 e 68,3 ± 6,5 anos; p= 0,006), índice de massa corporal (IMC) (23,1 ± 2,8 e 28,2 ± 4,2 kg/m^2^; p <0,001), e FEVE (29,9 ± 8,8 e 37,9 ± 12,1%; p= 0,005) eram diferentes nos grupos com e sem sarcopenia, respectivamente. Depois de normalizar em relação à idade, etnia, IMC, FEVE, e o uso de inibidores da enzima conversora de angiotensina/bloqueadores de receptor de angiotensina, a sarcopenia foi associada a níveis séricos de IL-6 mais altos e capacidade funcional pior.

**Conclusão:**

Em pacientes com IC, a sarcopenia foi associada aos níveis de IL-6 e à capacidade funcional.

## Introdução

A sarcopenia, uma alteração muscular progressiva e generalizada da musculatura esquelética, que está associada ao aumento da probabilidade de resultados adversos, tais como quedas, fraturas, incapacidade física e mortalidade,^1^ tem recebido cada vez mais atenção em pacientes com insuficiência cardíaca (IC) nos últimos anos.^2^

Já se reconhece que a sarcopenia tem uma importância clínica em relação à gravidade da IC e que as duas doenças podem interagir.^3^ A prevalência da sarcopenia na IC pode variar de acordo com estudo. De acordo com uma meta-análise recente, a prevalência combinada da sarcopenia na IC foi de 34%,^4^ mas ela pode chegar a 50% entre pacientes hospitalizados por piora da IC.^5^ Ela também está associada a um prognóstico desfavorável,^3^ contribuindo para a redução da capacidade de exercício,^6^ mortalidade global e cardiovascular mais alta, e aumento das repetições da hospitalização, bem como a perda da autonomia e uma qualidade de vida pior.^3 , 4^

A etiologia da sarcopenia é complexa e multifatorial,^7^ incluindo anormalidades endócrinas e metabólica, e tem interações próximas com a inflamação sistêmica de baixo grau em indivíduos idosos (inflammageing),^7 , 8^ redução da síntese e da regeneração da proteína, aumento da apoptose e lise de proteína.^8^ Nesse contexto, o desenvolvimento de possíveis biomarcadores especificamente relacionados a rotas fisiopatológicas diferentes, tais como a junção neuromuscular, fatores de crescimento, o sistema endócrino, renovação de proteínas e rotas comportamentais e inflamatórias, poderia ajudar a esclarecer os mecanismos fisiopatológicos da sarcopenia na IC.^8^

A inclusão da avaliação de sarcopenia na rotina clínica é essencial para o tratamento de pacientes com IC, já que a perda muscular nessa população é mais acelerada e acentuada,^4^ especialmente em adultos mais velhos. Embora alguns biomarcadores tenham sido sugeridos,^8^ esses aspectos ainda não foram elucidados em pacientes com IC. Considerando que a IC compartilha caminhos fisiopatológicos com a sarcopenia^9^ e a possibilidade de usar esses biomarcadores nesses pacientes, o objetivo desse estudo foi avaliar a associação entre marcadores inflamatórios e humorais e sarcopenia, bem como o impacto da sarcopenia na qualidade de vida de na capacidade funcional em pacientes com IC mais velhos.

## Métodos

**Figura 1:**
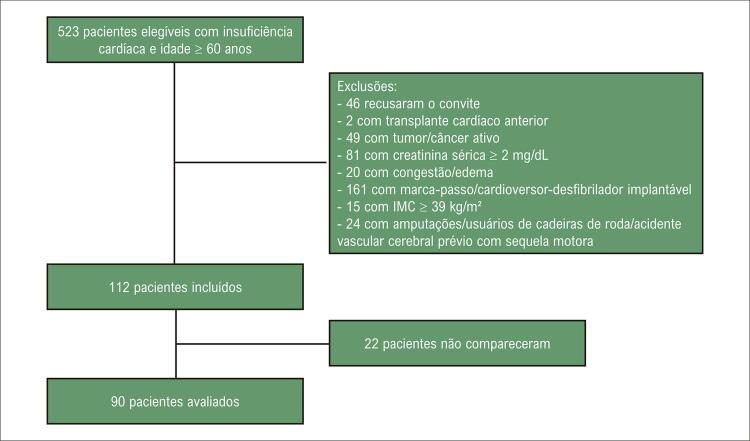
– Fluxograma de recrutamento de pacientes. IMC: índice de massa corporal.

A amostra deste estudo transversal foi composta por idosos com IC de ambos os sexos (idade ≥60 anos), com pelo menos 3 meses de diagnóstico de IC, classificados de acordo com a classe funcional da New York Heart Association e triados no ambulatório de insuficiência cardíaca de um hospital terciário do sul do Brasil, que foram recrutados consecutivamente ( Figura 1 ) entre março de 2018 e novembro de 2019. Os critérios de exclusão foram creatinina sérica ≥2,0 mg/dL, pacientes em terapia renal substitutiva, transplante cardíaco prévio, IC descompensada, congestão e/ou edema periférico avaliados em consulta médica, história de angina instável, tumores malignos ativos, infecção aguda, contraindicações para análise de bioimpedância elétrica (BIA) (como marca-passo ou cardioversor-desfibrilador implantável, pois no momento do planejamento do projeto ainda não havia evidências que corroboravam o uso seguro de BIA nesses indivíduos) e dificuldade de realizar testes funcionais (cadeirantes, amputados ou portadores de sequelas motoras de acidente vascular cerebral anterior).

### Características sociodemográficas, clínicas e antropométricas

Dados sociodemográficos, comorbidades, tratamento farmacológico, classe funcional da New York Heart Association, etiologia da IC e ecocardiograma bidimensional para obtenção do valor da fração de ejeção ventricular esquerda (FEVE) foram coletados dos prontuários e verificados durante a anamnese e consulta clínica do paciente.

Foi usada uma balança digital (Toledo®, Araçatuba, São Paulo, Brasil) para a pesagem dos pacientes, e um estadiômetro vertical (Veeder-Root® 2.0m, São Bernardo do Campo, São Paulo, Brasil) foi usado para medir sua altura. O IMC foi calculado e classificado de acordo com os pontos de corte recomendados para adultos mais velhos.^10^ Para calcular a circunferência muscular do braço (CMB), foi utilizada uma fita métrica não elástica (Cescorf Scientific, Cescorf, Brasil) para determinar a circunferência do braço. A espessura da dobra cutânea tricipital também foi medida. A partir do 50º percentil, a adequação da CMB foi calculada e classificada de acordo com o estado nutricional.^11^ Além disso, com o paciente na posição sentada, com a perna dobrada em um ângulo de 90° e os pés apoiados no chão, a circunferência da panturrilha da perna não dominante foi medida no ponto mais largo.^12^ Valores <31 cm foram considerados indicativos de massa muscular baixa.^13^

### Parâmetros inflamatórios e humorais

Os níveis séricos da proteína C reativa de alta sensibilidade (PCR-as), fator de crescimento semelhante à insulina tipo 1 (IGF-1), e testosterona total foram determinados por protocolos hospitalares padrão: o valor da PCR-as foi determinado pela análise de imunoturbidimetria, IGF-1, por quimioluminescência, e testosterona total, por imunoensaio competitivo por eletroquimioluminescência.

Para a análise da interleucina-6 (IL-6) e do fator de necrose tumoral alfa (TNF-α), amostras de sangue foram centrifugadas a 4 °C, 2500 rpm por 15 minutos para extração do soro e armazenadas a -80 °C for para análise posterior. Um kit de imunoensaio multiplex ProcartexPlex 2-plex Human Custom High-Sensitivity (Thermo Fisher Scientific®, Vienna, Áustria: número de catálogo PPXS-02-MXPRKP3) foi usado de acordo com as instruções do fabricante. As amostras passaram por um processo único de descongelamento (para a presente análise), e a parte não utilizada foi descartada.

### Classificação da sarcopenia

O riso de sarcopenia foi avaliado usando o questionário SARC-F.^14^ As categorias de sarcopenia provável, sarcopenia e sarcopenia grave foram definidas pelos critérios EWGSOP2, em que a sarcopenia provável é identificado pela presença de baixa força muscular; o diagnóstico de sarcopenia é confirmado pela presença de baixa força muscular e baixa quantidade ou qualidade de músculos; e a sarcopenia grave é identificada quando é detectada a presença de baixa força muscular, baixa quantidade ou qualidade de músculos e baixo desempenho físico.^1^

### Força muscular

A força muscular foi avaliada pelo teste de força de preensão manual^15^ e pelo teste de sentar e levantar cinco vezes.^16^ O teste de força de preensão manual foi realizado com um dinamômetro mecânico Jamar® (Sammons Preston Rolyan, Bolingbrook, IL, EUA), com o paciente sentado com apoio para as costas, sem apoio para o braço, e com o cotovelo dobrado a 90°.^17^ O teste foi repetido três vezes com a mão dominante e o valor mais alto das três medições foi usado.^18^ Os pontos de corte EWGSOP2 foram usados (baixa força muscular: valores <27 kgf para homens e <16 kgf para mulheres).^1^

O teste de sentar e levantar cinco vezes mede o tempo necessário para um indivíduo levantar cinco vezes da posição sentada sem usar os braços. Os participantes foram instruídos a cruzar os braços sobre o peito e se levantar da cadeira uma vez. Se eles realizassem o movimento com sucesso, eles eram instruídos a repetir a manobra cinco vezes em seguida o mais rápido possível sem parar.^16^ O ponto de corte EWGSOP2 foi usado (baixa força muscular: tempo de teste >15 segundos).^1^

### Massa muscular

A massa muscular foi estimada usando-se uma equação preditiva.^19^ Para obter dados de bioimpedância elétrica, foi usado um analisador de bioimpedância Biodynamics BIA 450 (800mA, 50 kHz; Biodynamics Corporation, Seattle, Washington, EUA), seguindo protocolos padrão.^20^ Os valores EWGSOP2 foram usados para classificar a massa muscular (baixa massa muscular: massa muscular esquelética apendicular (MMEA) abaixo de 20 kg para homens e abaixo de 15 kg para mulheres). Os valores da MMEA também foram normalizados para a altura (MMEA/altura^2^), e a massa muscular baixa foi considerada <7,0 kg/m^2^ e <5,5 kg/m^2^ para homens e mulheres, respectivamente.^1^

### Desempenho físico

O desempenho físico foi avaliado pelo teste de velocidade de marcha de 6 metros. O paciente foi cronometrado enquanto andava em seu ritmo usual em um trajeto de 6 metros em uma linha reta marcada no chão.^16^ O teste foi aplicado duas vezes. O tempo mais rápido dos dois foi usado e um ponto de corte de ≤0,8 m/s foi considerado baixo desempenho físico.^1^

### Nível de atividade física

O nível de atividade física do paciente foi avaliado e classificado usando o Questionário internacional de atividade física - versão curta.^21^

### Capacidade funcional

A capacidade funcional foi medida com o teste de caminhada de 6 minutos de acordo com um protocolo padronizado.^22^ Uma distância de menos de 300 metros foi caracterizada como mau desempenho para pacientes com IC.^23^

### Qualidade de vida

A qualidade de vida foi avaliada usando a versão validade em português do *Minnesota Living with Heart Failure Questionnaire.* ^24^ A pontuação total variou entre 0 e 105 pontos, e as pontuações mais altas indicavam uma qualidade de vida mais baixa.

### Análise estatística

O cálculo do tamanho da amostra foi realizado usando o WinPEPI (Programs for Epidemiologists for Windows), versão 11.43, com base nos estudos conduzidos por Onoue et al. (2016) e Harada et al. (2017).^25 , 26^ Considerando um nível de significância de 5%, um poder de 80%, uma prevalência de sarcopenia estimada em 20% e um tamanho de efeito mínimo de 0,8 desvios padrão entre os grupos em relação aos parâmetros de PCR, TNF-α, IGF-1, e testosterona, chegou-se a um mínimo total de 90 pacientes.

As variáveis quantitativas foram descritas como média e desvio padrão (DP), ou mediana e intervalo interquartil, de acordo com a normalidade dos dados. O teste de Shapiro-Wilk foi usado para determinar a normalidade. As variáveis categóricas foram descritas por frequências absolutas e relativas.

Para comparar as médias, foi aplicado o Teste t de Student para amostras independentes. Em caso de assimetria, o teste Mann-Whitney foi utilizado. Ao comparar as proporções, aplicou-se o qui-quadrado de Pearson ou teste exato de Fisher. No caso da significância estatística, a análise residual normalizada foi usada para localizar as associações.

Para controlar fatores de confusão, as análises de regressão de Poisson univariada e multivariada foram utilizadas. Variáveis com um p-valor <0,10 na análise univariada foram incluídas no modelo multivariado. O nível de significância foi definido em 5% (p<0,05), e as análises foram realizadas no SPSS 21.0.

## Resultados

Foram incluídos noventa pacientes com IC, 67,8% do sexo masculino, com uma média de idade de 69,4 ± 7,2 anos. As classes funcionais I e II da New York Heart (77,8%) e a etiologia não isquêmica (71,1%) predominaram, com uma FEVE média de 35,9 ± 11,9%. Em relação ao tratamento farmacológico, 94,4% dos pacientes foram tratados com betabloqueadores, e 93,3% foram tratados com inibidores da enzima conversora da angiotensina (IECA)/bloqueadores de receptor de angiotensina (BRA) ( Tabela 1 ).

**Tabela 1:** – Características demográficas, clínicas e nutricionais, nível de atividade física, e qualidade de vida em pacientes com insuficiência cardíaca com ou sem sarcopenia

	Todos os pacientes (n = 90)	Sem sarcopenia (n = 68)	Com sarcopenia (n = 22)	p
Masculino	61 (67,8)	46 (67,6)	15 (68,2)	1,000
Idade (anos)	69,4 ± 7,2	68,3 ± 6,5	73,1 ± 8,1	0,006
**Raça**				
Brancos	70 (77,8)	50 (73,5)	20 (90,9)	
Não brancos	20 (22,2)	18 (26,5)	2 (9,1)	0,139
**Etiologia da insuficiência cardíaca**				
Isquêmica	26 (28,9)	19 (27,9)	7 (31,8)	0,584
Não isquêmica	24 (26,7)	20 (29,4)	4 (19,2)	
Hipertensos	40 (44,4)	29 (42,6)	11 (50,0)	
FEVE (%)	35,9 ± 11,9	37,9 ± 12,1	29,9 ± 8,8	0,005
**Classificação NYHA**				
I e II	70 (77,8)	55 (80,9)	15 (68,2)	0,244
III e IV	20 (22,2)	13 (19,1)	7 (31,8)	
ICFER	80 (88,9)	59 (86,8)	21 (95,5)	0,441
ICFEP	10 (11,1)	9 (13,2)	1 (4,5)	
**Medicamentos**				
IECA/BRA	84 (93,3)	67 (98,5)	17 (77,3)	0,003
Betabloqueadores	85 (94,4)	65 (95,6)	20 (90,9)	0,592
Digitálicos	28 (31,1)	20 (29,4)	8 (36,4)	0,728
Diuréticos	83 (92,2)	62 (91,2)	21 (95,5)	1,000
Peso (kg)	72,4 ± 14,5	76,9 ± 13,6	58,6 ± 5,8	<0,001
IMC (kg/m^2^)	26,9 ± 4,5	28,2 ± 4,2	23,1 ± 2,8	<0,001
**Classificação do IMC**				
Peso baixo	13 (14,4)	5 (7,4)	8 (36,4)*	<0,001
Eutrófico	31 (34,4)	20 (29,4)	11 (50,0)	
Sobrepeso	46 (51,1)	43 (63,2)*	3 (13,6)	
**Classificação da CP**				
<31 cm	3 (3,3)	0 (0,0)	3 (13,6)	0,013
≥31 cm	87 (96,7)	68 (100)	19 (86,4)	
**Classificação da CMB**				
Desnutrição	13 (14,4)	3 (4,4)	10 (45,5)	<0,001
Eutrofia	77 (85,6)	65 (95,6)	12 (54,5)	
**Comorbidades**				
HAS	63 (70,0)	50 (73,5)	13 (59,1)	0,309
Diabetes mellitus	33 (36,7)	25 (36,8)	8 (36,4)	1,000
Dislipidemia	12 (13,3)	9 (13,2)	3 (13,6)	1,000
**Nível de atividade física**				
Sedentário	22 (24,4)	16 (23,5)	6 (27,3)	0,931
Atividade irregular A/B	56 (62,2)	43 (63,2)	13 (59,1)	
Ativo	12 (13,3)	9 (13,2)	3 (13,6)	
Teste de caminhada de seis minutos	366,7 ± 88,9	383,1 ± 78,9	316,4 ± 100,9	0,002
**Qualidade de vida**				
Pontuação total no MLHFQ	23 (10 - 44)	19,5 (10 - 42,25)	37,5 (19,5 - 57,5)	0,033

Os dados foram expressos como %, n (%), média ± DP ou mediana e intervalo interquartil (P25-75). *Associação estatisticamente significativa pelo teste residual normalizado em 5% de significância. IECA: enzima conversora da angiotensina inibidores; BRA: bloqueadores de receptor de angiotensina; CMB: circunferência do músculo do braço; IMC: índice de massa corporal; CP: circunferência da panturrilha; IPAQ-s: Questionário internacional de atividade física - versão curta; ICFEP: insuficiência cardíaca com fração de ejeção preservada; ICFER: insuficiência cardíaca com fração de ejeção reduzida; FEVE: fração de ejeção ventricular esquerda; MLHFQ: Minnesota Living with Heart Failure Questionnaire; NYHA: New York Heart Association; HAS: hipertensão arterial sistêmica.

**Figura 2:**
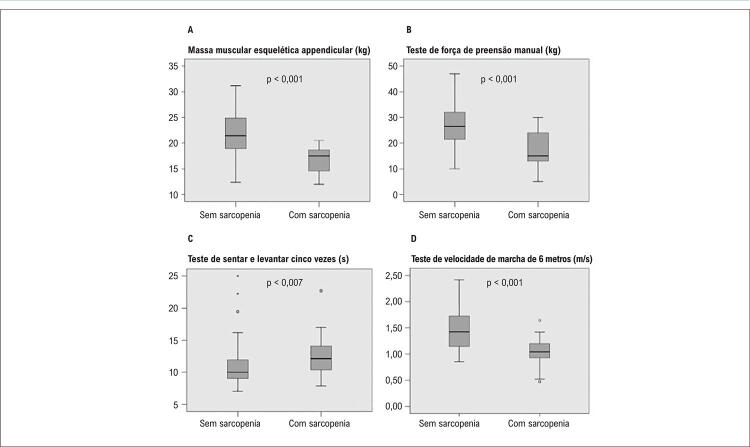
– Gráfico de caixa de critérios diagnósticos para sarcopenia. (A) Massa muscular esquelética apendicular em pacientes sem e com sarcopenia (21,44 kg (18,82 - 24,89) vs. 17,47 kg (14,29 - 18,66) (B) Força de preensão manual em pacientes sem e com sarcopenia (26,50 kg (21,25 - 32) vs. 15 kg (12,75 - 24), (C) Teste de sentar e levantar cinco vezes em pacientes sem e com sarcopenia (10 s (9,03 - 11,93) vs. 12,12 s (10,38 - 14,33), (D) teste de velocidade de marcha de 6 metros em pacientes sem e com sarcopenia (1,42 s (1,14 - 1,72) vs. 1,04 s (0,91 - 1,21).

Um risco de sarcopenia foi identificado em 35 (38,9%) pacientes, sarcopenia provável, em 39 (43,3%), sarcopenia, em 22 (24,4%), e sarcopenia grave em 4 (4,4%). Os valores médios para massa muscular, força muscular e desempenho físico são apresentados na Figura 2 .

Em relação aos marcadores humorais e inflamatórios, não houve diferença significativa nas médias para os níveis séricos de PCR-as, IL-6, TNF-α, IGF-1 ou testosterona total entre os grupos com e sem sarcopenia ( Tabela 2 ).

**Tabela 2:** – Sarcopenia e marcadores inflamatórios e humorais em pacientes com insuficiência cardíaca

	Sem sarcopenia (n = 68)	Com sarcopenia (n = 22)	p
PCR-as (mg/dL)	3,23 (1,78 - 6,42)	1,42 (0,81 - 10,8)	0,467
IGF-1 (ng/ml)	143,3 (97,9 - 177,3)	111,5 (87,8 - 165,4)	0,134
**Testosterona total (ng/ml)**			
Feminino	0,11 (0,04 - 0,19)	0,02 (0,02 - 0,13)	0,304
Masculino	4,02 ± 1,49	4,16 ± 1,71	0,757
IL-6 (pg/mL)	1,49 (0,85 - 2,32)	2,26 (0,92 - 3,78)	0,062
TNF-α (pg/mL)	0,62 (0,48 - 1,04)	0,72 (0,47 - 1,22)	0,538

Dados expressos como média ± desvio padrão ou mediana e P25 - P75. IGF-1: fator de crescimento semelhante à insulina tipo 1; IL-6: interleucina-6; mg/L: miligramas por litro; ng/ml: nanogramas por mililitro; pg/mL: picogramas por mililitro; TNF-α: fator de necrose tumoral alfa; PCR-as: proteína C reativa de alta sensibilidade.

Na análise univariada, a sarcopenia foi associada aos níveis séricos de IL-6 (p <0,001), assim como foram os resultados do teste de caminhada de 6 minutos (p = 0,012) ( Tabela 3 ). Para controlar a multicolinearidade, dois modelos multivariados foram realizados, o modelo 1 contendo interleucina-6 e outras variáveis, e o modelo 2, contendo o teste de caminhada de 6 minutos e outras variáveis. No modelo multivariado 1, após padronização para idade, IMC, etnia, FEVE e uso de IECA/BRA, a IL-6 permaneceu associada à sarcopenia: para cada aumento de 1 pg/mL na IL-6, houve um aumento de 10% na prevalência de sarcopenia. No modelo multivariado 2, após a normalização para idade, IMC, etnia, FEVE e uso de IECA/BRA, o teste de caminhada de 6 minutos também se mostrou significativamente associado à sarcopenia, o que demonstra que o baixo desempenho nesse teste leva a um aumento de 3 vezes na probabilidade de ter sarcopenia ( Tabela 3 ). Em relação ao IMC e FEVE, para cada aumento de uma unidade, houve uma diminuição de 22% e 4%, respectivamente, na prevalência de sarcopenia no modelo 1. No modelo 2, apenas o IMC permaneceu estatisticamente significativo, enquanto o aumento de uma unidade resultou em uma redução de 23% na prevalência de sarcopenia.

**Tabela 3:** – Análise univariada e multivariada entre sarcopenia e biomarcadores, atividade física e capacidade funcional (regressão de Poisson com estimador de erro robusto), por meio de dois modelos multivariados

Variáveis	Univariada		Modelo 1		Modelo 2	
	RP (IC 95%)	p	RP normalizado (IC 95%)	p	RP normalizado (IC 95%)	p
Idade (anos)	1,06 (1,02 – 1,11)	0,003	1,03 (0,98 – 1,09)	0,255	1,03 (0,98 – 1,08)	0,292
IMC (kg/m^2^)	0,79 (0,72 – 0,85)	<0,001	0,78 (0,70 – 0,87)	<0,001	0,77 (0,70 – 0,84)	<0,001
**Raça (%)**						
Brancos	2,86 (0,73 – 11,2)	0,132	-	-	-	-
Não brancos	1,00					
FEVE (%)	0,95 (0,92 – 0,98)	0,001	0,96 (0,94 – 0,99)	0,007	0,97 (0,94 – 1,00)	0,060
Uso de IECA/BRA	0,24 (0,14 – 0,42)	<0,001	0,60 (0,30 – 1,18)	0,141	0,95 (0,39 – 2,35)	0,919
PCR-as (mg/dL)	0,99 (0,96 – 1,03)	0,751	-	-	-	-
IGF-1 (mg/ml)	0,99 (0,98 - 1,00)	0,297	-	-	-	-
Testosterona total (ng/ml)	1,02 (0,86 - 1,20)	0,852	-	-	-	-
IL-6 (pg/mL)	1,15 (1,07 - 1,24)	<0,001	1,10 (1,02 - 1,18)	0,009	-	-
TNF-α (pg/mL)	1,08 (0,67 - 1,75)	0,752	-	-	-	-
**IPAQ-s**						
Sedentário	1,09 (0,27 - 4,36)	0,902	-	-		
Atividade irregular B/A	0,93 (0,27 - 3,26)	0,908	-	-		
Ativo	1,00		-	-		
**Teste de caminhada de seis minutos**						
Desempenho normal	1,00		-	-	1,00	
Desempenho baixo	2,97 (1,27 - 6,96)	0,012	-	-	3,06 (1,50 – 6,26)	0,002

IECA: enzima conversora da angiotensina inibidores; BRA: bloqueadores de receptor de angiotensina; IMC: índice de massa corporal; IGF-1: fator de crescimento semelhante à insulina tipo 1; IL-6: interleucina-6; IPAQ-s: Questionário internacional de atividade física - versão curta; FEVE: Fração de ejeção ventricular esquerda; TNF-α: fator de necrose tumoral alfa; PCR-as: proteína C reativa de alta sensibilidade; RP: razão de prevalência. Modelo 1: idade, IMC, FEVE, IECA/BRA, e interleucina-6; Modelo 2: idade, IMC, FEVE, IECA/BRA, e teste de caminhada de 6 minutos.

## Discussão

Os principais achados deste estudo referem-se à associação entre sarcopenia e níveis séricos de IL-6, bem como com a capacidade funcional em pacientes idosos com IC ( Figura central ).

**Figura Central:**
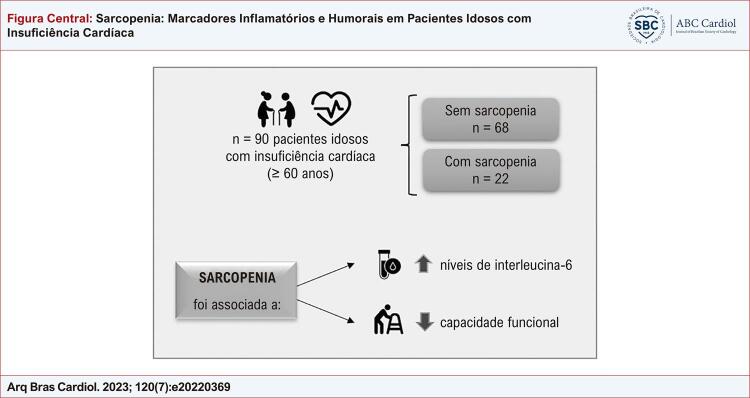
: Sarcopenia: Marcadores Inflamatórios e Humorais em Pacientes Idosos com Insuficiência Cardíaca

Resumo dos resultados principais.

A sarcopenia, além de altamente prevalente, foi associada a níveis mais elevados de interleucina-6 e diminuição da capacidade funcional.

A prevalência da sarcopenia foi mais próxima da identificada nos pacientes ambulatoriais do estudo SICA-HF,^27^ mas foi mais alta do que a identificada nos resultados de Canteri et al. (2019).^28^ Métodos diferentes de avaliação de MMEA, bem como níveis diferentes de atividade física, podem ter afetado essa diferença. O presente estudo reitera a associação entre sarcopenia e idade em pacientes com IC encontrada em outros estudos,^29 , 28^ bem como a associação entre sarcopenia e valores de FEVE mais baixos.^29^

Sabe-se que a IC pode induzir a sarcopenia por caminhos fisiopatológicos comuns em que um influencia o outro.^8^ A inflamação é um processo central na IC,^30^ considerando que pacientes com IC geralmente têm níveis baixos de inflamação sistêmica crônica, que pode ter um efeito contínuo nos músculos esqueléticos.^3^ Altos níveis de marcadores inflamatórios, tais como TNF-α, PCR-ase e IL-6, estão relacionados a uma diminuição da massa muscular e da força,^3 , 31^ que sugere que a inflamação, que também está envolvida na patogênese da sarcopenia, representa um elo essencial entre essas duas doenças.^9^

A IL-6, um dos marcadores inflamatórios relacionados à sarcopenia no presente estudo, já demonstrou estar associado a força e função muscular,^32^ além de já ter demonstrado uma associação com o prognóstico dessa população.^30^ Em doenças crônicas em adultos idosos, a IL-6 parece estar profundamente implicada na fisiopatologia da capacidade funcional reduzida, que leva à hipótese de que sua desregulação possa ser o primeiro passo no desenvolvimento da sarcopenia.^33^ Os níveis de TNF-α e IL-6 aumentados após o início da sarcopenia em adultos idosos residentes na comunidade corroboram essa hipótese.^34^ Entretanto, isso ainda é controverso na literatura,^35 , 31^ e, considerando o desenho transversal do presente estudo, somente hipóteses sobre o papel causal da IL-6 na sarcopenia em pacientes com IC podem ser especuladas.

O papel dos marcadores inflamatórios também pode estar relacionado à redução dos hormônios anabólicos descritos na sarcopenia. Neste estudo, níveis séricos de IGF-1 mais baixos não foram observados em pacientes sarcopênicos, apesar da associação com a IL-6. Um dos mecanismos pelos quais a IL-6 está ligada à sarcopenia é a interferência direta na transdução de sinal da insulina e inibição da produção e da atividade biológica do IGF-1.^36^ No presente estudo, nenhum dos marcadores do sistema endócrino foi associado à sarcopenia, o que pode sugerir que, devido a patologias comuns, os efeitos específicos da sarcopenia sobre os hormônios anabólicos podem não ser percebidos. Outra possível razão pode ser que o IGF-1 e a testosterona terem sido considerados apenas variáveis contínuas em nosso estudo, enquanto a presença de deficiência de testosterona e/ou síndrome de baixo IGF-1 não foi investigada.^37^

Além das diferenças significativas nos valores médios dos componentes individuais da sarcopenia entre pacientes com e sem sarcopenia, o grupo com sarcopenia também teve pior desempenho no teste de caminhada de 6 minutos, um parâmetro de capacidade de exercício bem estabelecido com valor prognóstico para mortalidade em pacientes com IC estável.^23^ A literatura relata que déficits em massa muscular, força e capacidade de exercício contribuem para reduzir a qualidade de vida de pacientes com IC.^3 , 4^ No presente estudo, a capacidade funcional foi associada à sarcopenia, o que indica um efeito sinérgico provável entre as duas doenças e seu efeito na capacidade funcional.

Outro problema importante a ser considerado em estudos relacionados à sarcopenia em pacientes com IC é o tratamento farmacológico. Alguns medicamentos padrão para IC demonstraram benefícios potenciais contra a perda muscular. No presente estudo, a maioria dos indivíduos (acima de 90%) tinham tratamento farmacológico otimizado na análise geral, o que pode ter afetado a prevalência da sarcopenia na amostra.

Além disso, a identificação precoce da sarcopenia nessa população e a implementação de estratégias terapêuticas voltadas para a recuperação da massa e função muscular podem contribuir para um melhor manejo clínico desses indivíduos, a fim de prevenir desfechos negativos à saúde.

### Limitações

Uma das limitações deste estudo reside no fato de que pacientes com cardioversores-desfibriladores implantáveis/terapia de ressincronização cardíaca não foram incluídos devido a algumas restrições no uso de BIA, o que, hipoteticamente, poderia ter afetado a ausência de maiores associações com os biomarcadores estudados. Além disso, a natureza transversal do estudo limita conclusões sobre causalidade, embora os resultados da análise multivariada, que foram normalizados para fatores importantes como idade, IMC, etnia, FEVE e uso de IECA/BRA, reforcem a ideia de que a IL-6 possa servir como um marcador de sarcopenia nesses pacientes. Outro aspecto é o número limitado de pacientes com sarcopenia neste estudo. Outro aspecto positivo do estudo foi a inclusão de vários parâmetros inflamatórios e hormonais, já que a natureza multifatorial da etiologia da sarcopenia na IC e a complexa interação entre as duas condições provavelmente requerem uma abordagem multidimensional. Este estudo também apresenta uma avaliação precisa e extensa sobre a sarcopenia em pacientes com IC, o que pode ajudar na detecção e prevenção precoce dessa doença e orientar as principais abordagens terapêuticas.

Diante dos achados identificados neste estudo, fica clara a importância de incluir a avaliação da sarcopenia na rotina clínica dessa população, já que a sarcopenia está diretamente relacionada ao prognóstico e ao avanço da IC e torna-se fundamental para o manejo desses pacientes.

Nosso estudo teve como objetivo avaliar a sarcopenia e sua associação com marcadores inflamatórios e humorais, qualidade de vida e capacidade funcional em pacientes idosos com IC. Assim, acreditamos que nossos resultados podem contribuir significativamente para um melhor entendimento dessa complexa relação, podendo oferecer uma base preliminar para prevenção, diagnóstico e tratamento da sarcopenia em pacientes com IC.

## Conclusões

Em suma, este estudo demonstrou que a sarcopenia é altamente prevalente e está associada a níveis mais elevados de IL-6 e redução da capacidade funcional (de acordo com o teste da caminhada de 6 minutos) em pacientes idosos com IC. Os resultados sugerem que pelo menos um dos parâmetros inflamatórios estudados pode estar relacionado à redução da força e da massa muscular em pacientes idosos com IC.

## References

[1] Cruz-Jentoft AJ, Bahat G, Bauer J, Boirie Y, Bruyère O, Cederholm T (2019). Sarcopenia: Revised European Consensus on Definition and Diagnosis. Age Ageing.

[2] von Haehling S (2018). Muscle Wasting and Sarcopenia in Heart Failure: A Brief Overview of the Current Literature. ESC Heart Fail.

[3] Yin J, Lu X, Qian Z, Xu W, Zhou X (2019). New Insights Into the Pathogenesis and Treatment of Sarcopenia in Chronic Heart Failure. Theranostics.

[4] Zhang Y, Zhang J, Ni W, Yuan X, Zhang H, Li P (2021). Sarcopenia in Heart Failure: A Systematic Review and Meta-Analysis. ESC Heart Fail.

[5] Reeves GR, Pandey A, Kitzman DW (2021). The Other Striated Muscle: The Role of Sarcopenia in Older Persons with Heart Failure. J Am Geriatr Soc.

[6] Bekfani T, Pellicori P, Morris DA, Ebner N, Valentova M, Steinbeck L (2016). Sarcopenia in Patients with Heart Failure with Preserved Ejection Fraction: Impact on Muscle Strength, Exercise Capacity and Quality of Life. Int J Cardiol.

[7] Morley JE, Anker SD, von Haehling S (2014). Prevalence, Incidence, and Clinical Impact of Sarcopenia: Facts, Numbers, and Epidemiology-Update 2014. J Cachexia Sarcopenia Muscle.

[8] Curcio F, Ferro G, Basile C, Liguori I, Parrella P, Pirozzi F (2016). Biomarkers in Sarcopenia: A Multifactorial Approach. Exp Gerontol.

[9] Collamati A, Marzetti E, Calvani R, Tosato M, D’Angelo E, Sisto AN (2016). Sarcopenia in Heart Failure: Mechanisms and Therapeutic Strategies. J Geriatr Cardiol.

[10] Lipschitz DA (1994). Screening for Nutritional Status in the Elderly. Prim Care.

[11] Blackburn GL, Thornton PA (1979). Nutritional Assessment of the Hospitalized Patient. Med Clin North Am.

[12] Onis M, Habicht JP (1996). Anthropometric Reference Data for International Use: Recommendations from a World Health Organization Expert Committee. Am J Clin Nutr.

[13] Landi F, Onder G, Russo A, Liperoti R, Tosato M, Martone AM (2014). Calf Circumference, Frailty and Physical Performance among Older Adults Living in the Community. Clin Nutr.

[14] Malmstrom TK, Miller DK, Simonsick EM, Ferrucci L, Morley JE (2016). SARC-F: A Symptom Score to Predict Persons with Sarcopenia at Risk for Poor Functional Outcomes. J Cachexia Sarcopenia Muscle.

[15] Roberts HC, Denison HJ, Martin HJ, Patel HP, Syddall H, Cooper C (2011). A Review of the Measurement of Grip Strength in Clinical and Epidemiological Studies: Towards a Standardised Approach. Age Ageing.

[16] Cesari M, Kritchevsky SB, Newman AB, Simonsick EM, Harris TB, Penninx BW (2009). Added Value of Physical Performance Measures in Predicting Adverse Health-Related Events: Results from the Health, Aging and Body Composition Study. J Am Geriatr Soc.

[17] Hillman TE, Nunes QM, Hornby ST, Stanga Z, Neal KR, Rowlands BJ (2005). A Practical Posture for Hand Grip Dynamometry in the Clinical Setting. Clin Nutr.

[18] Schlüssel MM, Anjos LA, Vasconcellos MT, Kac G (2008). Reference Values of Handgrip Dynamometry of Healthy Adults: A Population-Based Study. Clin Nutr.

[19] Kyle UG, Genton L, Hans D, Pichard C (2003). Validation of a Bioelectrical Impedance Analysis Equation to Predict Appendicular Skeletal Muscle Mass (ASMM). Clin Nutr.

[20] Kyle UG, Bosaeus I, Lorenzo AD, Deurenberg P, Elia M, Gómez JM (2004). Bioelectrical Impedance Analysis-Part II: Utilization in Clinical Practice. Clin Nutr.

[21] Craig CL, Marshall AL, Sjöström M, Bauman AE, Booth ML, Ainsworth BE (2003). International Physical Activity Questionnaire: 12-Country Reliability and Validity. Med Sci Sports Exerc.

[22] ATS Committee on Proficiency Standards for Clinical Pulmonary Function Laboratories (2002). ATS Statement: Guidelines for the Six-Minute Walk Test. Am J Respir Crit Care Med.

[23] Rostagno C, Olivo G, Comeglio M, Boddi V, Banchelli M, Galanti G (2003). Prognostic Value of 6-Minute Walk Corridor Test in Patients with Mild to Moderate Heart Failure: Comparison with Other Methods of Functional Evaluation. Eur J Heart Fail.

[24] Carvalho VO, Guimarães GV, Carrara D, Bacal F, Bocchi EA (2009). Validation of the Portuguese Version of the Minnesota Living with Heart Failure Questionnaire. Arq Bras Cardiol.

[25] Onoue Y, Izumiya Y, Hanatani S, Tanaka T, Yamamura S, Kimura Y (2016). A Simple Sarcopenia Screening Test Predicts Future Adverse Events in Patients with Heart Failure. Int J Cardiol.

[26] Harada H, Kai H, Shibata R, Niiyama H, Nishiyama Y, Murohara T (2017). New Diagnostic Index for Sarcopenia in Patients with Cardiovascular Diseases. PLoS One.

[27] Emami A, Saitoh M, Valentova M, Sandek A, Evertz R, Ebner N (2018). Comparison of Sarcopenia and Cachexia in Men with Chronic Heart Failure: Results from the Studies Investigating Co-morbidities Aggravating Heart Failure (SICA-HF). Eur J Heart Fail.

[28] Canteri AL, Gusmon LB, Zanini AC, Nagano FE, Rabito EI, Petterle RR (2019). Sarcopenia in Heart Failure with Reduced Ejection Fraction. Am J Cardiovasc Dis.

[29] Fülster S, Tacke M, Sandek A, Ebner N, Tschöpe C, Doehner W (2013). Muscle Wasting in Patients with Chronic Heart Failure: Results from the Studies Investigating Co-Morbidities Aggravating Heart Failure (SICA-HF). Eur Heart J.

[30] Shirazi LF, Bissett J, Romeo F, Mehta JL (2017). Role of Inflammation in Heart Failure. Curr Atheroscler Rep.

[31] Markousis-Mavrogenis G, Tromp J, Ouwerkerk W, Devalaraja M, Anker SD, Cleland JG (2019). The Clinical Significance of Interleukin-6 in Heart Failure: Results from the BIOSTAT-CHF study. Eur J Heart Fail.

[32] Hanberg JS, Rao VS, Ahmad T, Chunara Z, Mahoney D, Jackson K (2018). Inflammation and Cardio-Renal Interactions in Heart Failure: A Potential Role for Interleukin-6. Eur J Heart Fail.

[33] Maggio M, Guralnik JM, Longo DL, Ferrucci L (2006). Interleukin-6 in Aging and Chronic Disease: A Magnificent Pathway. J Gerontol A Biol Sci Med Sci.

[34] Bian AL, Hu HY, Rong YD, Wang J, Wang JX, Zhou XZ (2017). A Study on Relationship between Elderly Sarcopenia and Inflammatory Factors IL-6 and TNF-α. Eur J Med Res.

[35] Bano G, Trevisan C, Carraro S, Solmi M, Luchini C, Stubbs B (2017). Inflammation and Sarcopenia: A Systematic Review and Meta-Analysis. Maturitas.

[36] Barbieri M, Ferrucci L, Ragno E, Corsi A, Bandinelli S, Bonafè M (2003). Chronic Inflammation and the Effect of IGF-I on Muscle Strength and Power in Older Persons. Am J Physiol Endocrinol Metab.

[37] Bossone E, Arcopinto M, Iacoviello M, Triggiani V, Cacciatore F, Maiello C (2018). Multiple Hormonal and Metabolic Deficiency Syndrome in Chronic heart Failure: Rationale, Design, and Demographic Characteristics of the T.O.S.CA. Registry. Intern Emerg Med.

