# Supplementary material for: Sarcopenia: Marcadores Inflamatórios e Humorais em Pacientes Idosos com Insuficiência Cardíaca
Source: Arq Bras Cardiol. 2023 Jul 27;120(7):e20220369. [Article in Portuguese] doi: 10.36660/abc.20220369 (PMC10382140; doi:10.36660/abc.20220369)
Supplement: Translation

# Translation: Sarcopenia: Inflammatory and Humoral Markers in Older Heart Failure Patients

**Authors:** Tamirys Delazeri Sangali, Gabriela Corrêa Souza, Édina Caroline Ternus Ribeiro, Ingrid Dalira Schweigert Perry

**Affiliations:** 1 Brazil Universidade Federal do Rio Grande do Sul, Porto Alegre, RS – Brazil; 2 Brazil Hospital de Clínicas de Porto Alegre, Porto Alegre, RS – Brazil

**Keywords:** Sarcopenia, Biomarkers, Inflammation, Heart Failure

## Abstract

**Background:**

Sarcopenia is highly prevalent in heart failure (HF) patients, and the involvement of biomarkers in its pathophysiology is suggested, but little has been studied concerning HF sarcopenic patients.

**Objectives:**

To evaluate the association between inflammatory and humoral markers with sarcopenia, as well as the impact of sarcopenia on quality of life and functional capacity in older HF patients.

**Methods:**

In this cross-sectional study, 90 outpatient HF patients, aged ≥ 60 years, were evaluated for sarcopenia (EWGSOP2 diagnostic criteria), inflammation (high-sensitive C-reactive protein [hs-CRP], Interleukin-6 [IL-6], tumor necrosis factor alpha [TNF-α]) and humoral markers (total testosterone and insulin-like growth factor-1 [IGF-1]), physical activity (International Physical Activity Questionnaire), quality of life (Minnesota Living with Heart Failure Questionnaire), and functional capacity (6-minute walk test). The adopted level of significance was p<0.05.

**Results:**

Patients had a mean age of 69.4 ± 7.2 years, 67.8% were male, with left ventricular ejection fraction (LVEF) of 35.9 ± 11.9% and 22 (24.4%) were sarcopenic. Age (73.1 ± 8.1 and 68.3 ± 6.5 years; p= 0.006), body mass index (BMI) (23.1 ± 2.8 and 28.2 ± 4.2 kg/m^2^; p <0.001), and LVEF (29.9 ± 8.8 and 37.9 ± 12.1%; p= 0.005) were different between groups with and without sarcopenia, respectively. After adjusting for age, ethnicity, BMI, LVEF, and the use of angiotensin converting enzyme inhibitors/angiotensin receptor blockers, sarcopenia was associated with higher serum levels of IL-6 and worse functional capacity.

**Conclusion:**

In HF patients, sarcopenia was associated with IL-6 levels and functional capacity.

## Introduction

Sarcopenia, a progressive and widespread muscle disorder of the skeletal musculature, which is associated with an increased likelihood of adverse outcomes, such as falls, fractures, physical disability, and mortality,^1^ has received increasing attention in patients with heart failure (HF) in recent years.^2^

It is recognized that sarcopenia has a clinical importance regarding HF severity and both conditions can interact.^3^ Prevalence of sarcopenia in HF can vary according to the study. According to a recent meta-analysis, the pooled prevalence of sarcopenia in HF was 34%,^4^ but it can be as high as 50% among patients admitted to worsening HF.^5^ It is also associated with an unfavorable prognosis,^3^ contributing to reduced exercise capacity,^6^ higher cardiovascular and all-cause mortality, and increased hospital readmissions, as well as the loss of autonomy and a lower quality of life.^3 , 4^

The etiology of sarcopenia is complex and multifactorial,^7^ including endocrine and metabolic abnormalities, and has close interactions with low-grade systemic inflammation in elderly individuals (inflammageing),^7 , 8^ reduced protein synthesis and regeneration, increased apoptosis, and lysis of protein.^8^ In this context, the development of potential biomarkers specifically related to different pathophysiological routes, such as the neuromuscular junction, growth factors, the endocrine system, protein turnover, and behavioral and inflammatory routes, could help clarify the pathophysiological mechanisms of sarcopenia in HF.^8^

Including sarcopenia assessment in the clinical routine is crucial for the management of HF patients, since muscle loss in this population is more accelerated and accentuated,^4^ especially in older adults. Although some biomarkers have been suggested,^8^ these aspects have not yet been elucidated in HF patients. Given that HF shares common pathophysiological pathways with sarcopenia^9^ and the possibility of using such biomarkers in these patients, the aim of this study was to evaluate the association between inflammatory and humoral markers with sarcopenia, as well as the impact of sarcopenia on the quality of life and functional capacity in older HF patients.

## Methods

The sample of this cross-sectional study consisted of older HF patients of both sexes (aged ≥60 years), with at least 3 months of HF diagnosis, classified according to New York Heart Association’s functional class and screened at the Heart Failure Outpatient Clinic from a tertiary hospital in southern Brazil, who were recruited consecutively ( Figure 1 ) between March 2018 and November 2019. The exclusion criteria were serum creatinine ≥2.0 mg/dL, subjects on renal replacement therapy, previous heart transplantation, decompensated HF, congestion and/or peripheral edema evaluated in a medical consultation, a history of uns. angina, active malignant tumors, acute infection, contraindications for electrical bioimpedance analysis (BIA) (such as a pacemaker or an implantable cardioverter-defibrillator, since at the time of project planning there was still no evidence to support the safe use of BIA in these subjects), and difficulty performing functional tests (wheelchair patients, amputees or those with motor sequelae from a previous stroke).

**Figure 1:** – Patient recruitment flowchart. BMI: body mass index.

### Sociodemographic, clinical, and anthropometric characteristics

Sociodemographic data, comorbidities, pharmacological treatment, New York Heart Association’s functional class, HF etiology, and two-dimensional echocardiography to obtain the left ventricular ejection fraction (LVEF) value were collected from the medical records and checked during the patient’s anamnesis and clinical consultation.

A digital scale (Toledo®, Araçatuba, São Paulo, Brazil) was used to measure the patients’ weight, and a vertical stadiometer (Veeder-Root® 2.0m, São Bernardo do Campo, São Paulo, Brazil) was used to measure their height. BMI was calculated and classified according to cutoff points recommended for older adults.^10^ To calculate the arm muscle circumference (AMC), a non-stretch tape measure (Cescorf Scientific, Cescorf, Brazil) was used to determine the arm circumference; tricipital skinfold thickness was also measured. Beginning with the 50th percentile, AMC adequacy was calculated and classified according to nutritional status.^11^ Additionally, with the patient in a sitting position, having the leg bent at a 90º angle and feet flat on the floor, the calf circumference of the non-dominant leg was measured at the widest point.^12^ Values <31 cm were considered indicative of low muscle mass.^13^

### Inflammatory and humoral parameters

Serum levels of high-sensitive C-reactive protein (hs-CRP), insulin-like growth factor-1 (IGF-1), and total testosterone were determined from standard hospital protocols: the value for hs-CRP was determined by immunoturbidimetry analysis, IGF-1 by chemiluminescence, and total testosterone by competitive electrochemiluminescence.

For Interleukin-6 (IL-6) and tumor necrosis factor alpha (TNF-α) analysis, blood samples were centrifuged at 4°C, 2500 rpm for 15 minutes for serum extraction and stored at -80°C for further analysis. A ProcartexPlex 2-plex Human Custom High-Sensitivity multiplex immunoassay kit (Thermo Fisher Scientific®, Vienna, Austria: catalog number PPXS-02-MXPRKP3) was used according to the manufacturer’s instructions. The samples went through a single defrosting process (for the present analysis), and the unused portion was discarded.

### Classification of sarcopenia

The risk of sarcopenia was assessed using the SARC-F questionnaire.^14^ Probable sarcopenia, sarcopenia, and severe sarcopenia were defined according to EWGSOP2 criteria, where probable sarcopenia is identified by the presence of low muscle strength; the diagnosis of sarcopenia is confirmed by the presence of low muscle strength and low muscle quantity or quality; and severe sarcopenia is identified when low muscle strength, low muscle quantity or quality, and low physical performance are detected.^1^

### Muscle strength

Muscle strength was assessed through handgrip strength^15^ and the Five-Times-Sit-to-Stand Test.^16^ Handgrip strength was measured using a Jamar® mechanical dynamometer (Sammons Preston Rolyan, Bolingbrook, IL, USA) while the patient was seated with back support, without arm support, and with the elbow flexed at 90º.^17^ The test was repeated three times with the dominant hand, and the highest value of the three measurements was used.^18^ EWGSOP2 cutoff points were used (low muscle strength: values <27 kgf for men and <16 kgf for women).^1^

The Five-Times-Sit-to-Stand Test measures the amount of time it takes for an individual to stand up five times from a sitting position without using their arms. The participants were asked to fold their arms over their chest and rise from the chair once. If they performed the movement successfully, they were instructed to repeat the maneuver five times in a row as quickly as possible without stopping.^16^ The EWGSOP2 cutoff point was used (low muscle strength: test run time >15 seconds).^1^

### Muscle mass

Muscle mass was estimated using a predictive equation.^19^ To obtain electrical bioimpedance data, a Biodynamics BIA 450 Bioimpedance Analyzer (800mA, 50 kHz; Biodynamics Corporation, Seattle, Washington, USA) was used, following standard protocols.^20^ EWGSOP2 values were used to classify muscle mass (low muscle mass: Appendicular Skeletal Muscle Mass (ASMM) below 20 kg for men and below 15 kg for women). ASMM values were also adjusted for height (ASMM/height^2^), and low muscle mass was considered <7.0 kg/m^2^and <5.5 kg/m^2^for men and women, respectively.^1^

### Physical performance

Physical performance was evaluated with the 6-Meter Walk Test. The patient was timed while walking in his or her usual gait on a 6-meter course along a straight line marked on the floor.^16^ The test was applied twice. The fastest time of the two tests was used, and a cutoff point ≤0.8 m/s was considered low physical performance.^1^

### Physical activity level

The patients’ physical activity level was assessed and classified using the International Physical Activity Questionnaire-short version.^21^

### Functional capacity

Functional capacity was measured with the 6-minute walk test according to a standardized protocol.^22^ A distance of less than 300 meters was characterized as poor performance for HF patients.^23^

### Quality of life

Quality of life was evaluated using the validated Portuguese version of the Minnesota Living with Heart Failure Questionnaire.^24^ The total score ranged from 0 to 105 points, with higher scores indicating a lower quality of life.

### Ethical approval

This study was approved by the institution’s Research Ethics Committee (81019917.1.0000.5327) on 03/07/2018, and followed the Declaration of Helsinki principles for research in human beings, with all participants providing written informed consent.

### Statistical analysis

The sample size calculation was performed using the WinPEPI (Programs for Epidemiologists for Windows), version 11.43, and based on the study by Onoue et al. (2016) and Harada et al. (2017).^25 , 26^ Considering a significance level of 5%, a power of 80%, a prevalence of sarcopenia estimated at 20% and a minimum effect size of 0.8 standard deviations between groups regarding the parameters of CRP, TNF-α, IGF-1, and testosterone, a minimum total of 90 patients was obtained.

Quantitative variables were described as mean and standard deviation (SD), or median and interquartile range, according to data normality. To evaluate normality, the Shapiro-Wilk test was used. Categorical variables were described as absolute and relative frequencies.

To compare means, the Student’s *t* -test for independent samples was applied. In case of asymmetry, the Mann-Whitney test was used. When comparing proportions, the Pearson chi-square or Fisher’s exact tests were applied. In the case of statistical significance, the adjusted residual analysis was used to locate the associations.

To control confounding factors, univariate and multivariate Poisson regression analysis was used. Variables with a p-value <0.10 in the univariate analysis were entered in the multivariate model. The significance level was set at 5% (p<0.05), and the analyses were performed in SPSS 21.0.

## Results

Ninety HF patients were included, 67.8% male, with a mean age of 69.4 ± 7.2 years. New York Heart Association functional classes I and II (77.8%) and non-ischemic etiology (71.1%) predominated, with a mean LVEF of 35.9 ± 11.9%. In relation to pharmacological treatment, 94.4% of patients were treated with beta-blockers and 93.3% were treated with angiotensin-converting enzyme inhibitors (ACEI)/angiotensin receptor blockers (ARB) ( Table 1 ).

**Table 1:** – Demographic, clinical, nutritional characteristics, physical activity level, and quality of life in heart failure patients with and without sarcopenia

	All patients (n = 90)	Without sarcopenia (n =68)	With sarcopenia (n = 22)	p
Male	61 (67.8)	46 (67.6)	15 (68.2)	1.000
Age (years)	69.4 ± 7.2	68.3 ± 6.5	73.1 ± 8.1	0.006
**Race**				
White	70 (77.8)	50 (73.5)	20 (90.9)	
Not white	20 (22.2)	18 (26.5)	2 (9.1)	0.139
**Heart Failure Etiology**				
Ischemic	26 (28.9)	19 (27.9)	7 (31.8)	0.584
Non-ischemic	24 (26.7)	20 (29.4)	4 (19.2)	
Hypertensive	40 (44.4)	29 (42.6)	11 (50.0)	
LVEF (%)	35.9 ± 11.9	37.9 ± 12.1	29.9 ± 8.8	0.005
**NYHA Classification**				
I and II	70 (77.8)	55 (80.9)	15 (68.2)	0.244
III and IV	20 (22.2)	13 (19.1)	7 (31.8)	
HFrEF	80 (88.9)	59 (86.8)	21 (95.5)	0.441
HFpEF	10 (11.1)	9 (13.2)	1 (4.5)	
**Medications**				
ACEI/ARB	84 (93.3)	67 (98.5)	17 (77.3)	0.003
Beta-blocker	85 (94.4)	65 (95.6)	20 (90.9)	0.592
Digitalis	28 (31.1)	20 (29.4)	8 (36.4)	0.728
Diuretics	83 (92.2)	62 (91.2)	21 (95.5)	1.000
Weight (kg)	72.4 ± 14.5	76.9 ± 13.6	58.6 ± 5.8	<0.001
BMI (kg/m^2^)	26.9 ± 4.5	28.2 ± 4.2	23.1 ± 2.8	<0.001
**BMI Classification**				
Low weight	13 (14.4)	5 (7.4)	8 (36.4)*	<0.001
Eutrophic	31 (34.4)	20 (29.4)	11 (50.0)	
Overweight	46 (51.1)	43 (63.2)*	3 (13.6)	
**CC Classification**				
<31 cm	3 (3.3)	0 (0.0)	3 (13.6)	0.013
≥31 cm	87 (96.7)	68 (100)	19 (86.4)	
**AMC Classification**				
Malnutrition	13 (14.4)	3 (4.4)	10 (45.5)	<0.001
Eutrophy	77 (85.6)	65 (95.6)	12 (54.5)	
**Comorbidities**				
SAH	63 (70.0)	50 (73.5)	13 (59.1)	0.309
Diabetes mellitus	33 (36.7)	25 (36.8)	8 (36.4)	1.000
Dyslipidemia	12 (13.3)	9 (13.2)	3 (13.6)	1.000
**Physical activity level**				
Sedentary	22 (24.4)	16 (23.5)	6 (27.3)	0.931
Irregularly active A/B	56 (62.2)	43 (63.2)	13 (59.1)	
Active	12 (13.3)	9 (13.2)	3 (13.6)	
Six-minute walk test	366.7 ± 88.9	383.1 ± 78.9	316.4 ± 100.9	0.002
**Quality of life**				
MLHFQ total score	23 (10 - 44)	19.5 (10 - 42.25)	37.5 (19.5 - 57.5)	0.033

Data were expressed as %, n (%), mean ± SD or median and interquartile range (P25-75). *Statistically significant association by the residual test adjusted to 5% significance. ACEI: angiotensin-converting enzyme inhibitors; ARB: angiotensin receptor blockers; AMC: muscle circumference of the arm BMI: Body Mass Index; CC: calf circumference; IPAQ-s: International Physical Activity Questionnaire-short version; HFpEF: Heart failure with preserved ejection fraction; HFrEF: Heart failure with reduced ejection fraction; LVEF: Left ventricular ejection fraction; MLHFQ: Minnesota Living with Heart Failure Questionnaire; NYHA: New York Heart Association; SAH: systemic arterial hypertension.

A risk of sarcopenia was identified in 35 (38.9%) patients, probable sarcopenia in 39 (43.3%), sarcopenia in 22 (24.4%), and severe sarcopenia in 4 (4.4%). The mean values for muscle mass, strength, and physical performance are shown in Figure 2 .

**Figure 2:** – Boxplot of diagnostic criteria for sarcopenia. (A) Appendicular skeletal muscle mass in patients without and with sarcopenia (21.44 kg (18.82-24.89) vs 17.47 kg (14.29-18.66) (B) Handgrip strength in patients without and with sarcopenia (26.50 kg (21.25-32) vs 15 kg 12.75-24), (C) Five-Times-Sit-to-Stand test in patients without and with sarcopenia (10 s (9.03-11.93) vs 12.12 s (10,38-14.33), (D) 6-meter walk test in patients without and with sarcopenia (1.42 s (1.14-1.72) vs 1.04 s (0.91-1.21).

In relation to humoral and inflammatory markers, there was no significant difference in means for serum levels of hs-CRP, IL-6, TNF-α, IGF-1, or total testosterone between the groups with and without sarcopenia ( Table 2 ).

**Table 2:** – Sarcopenia and inflammatory and humoral markers in heart failure patients

	Without sarcopenia (n = 68)	With sarcopenia (n = 22)	p
hs-CRP (mg/L)	3.23 (1.78 - 6.42)	1.42 (0.81 - 10.8)	0.467
IGF-1 (ng/ml)	143.3 (97.9 - 177.3)	111.5 (87.8 - 165.4)	0.134
**Total testosterone (ng/ml)**			
Female	0.11 (0.04 - 0.19)	0.02 (0.02 - 0.13)	0.304
Male	4.02 ± 1.49	4.16 ± 1.71	0.757
IL-6 (pg/mL)	1.49 (0.85 - 2.32)	2.26 (0.92 - 3.78)	0.062
TNF-α (pg/mL)	0.62 (0.48 - 1.04)	0.72 (0.47 - 1.22)	0.538

Data expressed as mean±standard deviation or median and P25 - P75. IGF-1: insulin-like growth factor-1; IL-6: interleukin-6; mg/L: milligrams per liter; ng/ml: nanograms per milliliter; pg/mL: picograms per milliliter; TNF-α: tumor necrosis factor alpha; hs-CRP: high-sensitive C-reactive protein.

In the univariate analysis, sarcopenia was associated with serum IL-6 levels (p <0.001), as were the 6-minute walk test results (p = 0.012) ( Table 3 ). To control for multicollinearity, two multivariate models were performed, the model 1 containing interleukin-6 and other variables, and model 2 containing the 6-minute walk test and other variables. In the multivariate model 1, after adjusting for age, BMI, ethnicity, LVEF, and ACEI/ARB use, IL-6 remained associated with sarcopenia: for each 1 pg/mL increase in IL-6, there was an increase of 10% in sarcopenia prevalence. In multivariate model 2, after adjusting for age, BMI, ethnicity, LVEF, and ACEI/ARB use, the 6-minute walk test also proved to be significantly associated with sarcopenia, which demonstrates that having a low performance on this test leads to a 3-fold increase in the probability of having sarcopenia ( Table 3 ). In relation to BMI and LVEF, for each one unit increase, there was a 22% and 4% decrease, respectively, in sarcopenia prevalence in model 1. In model 2, only BMI remained statistically significant, whereas an increase of one unit resulted in a 23% reduction in the prevalence of sarcopenia.

**Table 3:** – Univariate and multivariate analysis between sarcopenia and biomarkers, physical activity, and functional capacity (Poisson regression with a robust error estimator), through two multivariate models

Variables	Univariate		Model 1		Model 2	
	PR (95% CI)	p	PR adjusted (95% CI)	p	PR adjusted (95% CI)	p
Age (years)	1.06 (1.02 – 1.11)	0.003	1.03 (0.98 – 1.09)	0.255	1.03 (0.98 – 1.08)	0.292
BMI (kg/m^2^)	0.79 (0.72 – 0.85)	<0.001	0.78 (0.70 – 0.87)	<0.001	0.77 (0.70 – 0.84)	<0.001
**Race (%)**						
White	2.86 (0.73 – 11.2)	0.132	-	-	-	-
Not white	1.00					
LVEF (%)	0.95 (0.92 – 0.98)	0.001	0.96 (0.94 – 0.99)	0.007	0.97 (0.94 – 1.00)	0.060
Use of ACEI/ARB	0.24 (0.14 – 0.42)	<0.001	0.60 (0.30 – 1.18)	0.141	0.95 (0.39 – 2.35)	0.919
hs-CRP (mg/L)	0.99 (0.96 – 1.03)	0.751	-	-	-	-
IGF-1 (mg/ml)	0.99 (0.98 - 1.00)	0.297	-	-	-	-
Total testosterone (ng/ml)	1.02 (0.86 - 1.20)	0.852	-	-	-	-
IL-6 (pg/mL)	1.15 (1.07 - 1.24)	<0.001	1.10 (1.02 - 1.18)	0.009	-	-
TNF-α (pg/mL)	1.08 (0.67 - 1.75)	0.752	-	-	-	-
**IPAQ-s**						
Sedentary	1.09 (0.27 - 4.36)	0.902	-	-		
Irregularly active B/A	0.93 (0.27 - 3.26)	0.908	-	-		
Active	1.00		-	-		
**Six-minute walk test**						
Normal performance	1.00		-	-	1.00	
Low performance	2.97 (1.27 - 6.96)	0.012	-	-	3.06 (1.50 – 6.26)	0.002

ACEI: angiotensin-converting enzyme inhibitors; ARB: angiotensin receptor blockers; BMI: Body Mass Index; IGF-1: insulin-like growth factor-1; IL-6: interleukin-6; IPAQ-s: International Physical Activity Questionnaire-short version; LVEF: Left ventricular ejection fraction; TNF-α: tumor necrosis factor alpha; hs-CRP: high-sensitive C-reactive protein. Model 1: age, BMI, LVEF, ACEI/ARB, and interleukin-6; Model 2: age, BMI, LVEF, ACEI/ARB, and 6-minute walk test.

## Discussion

The main findings of this study refer to the association between sarcopenia and serum levels of IL-6, as well as with functional capacity in older HF patients ( Central illustration ).

**Central Illustration:** : Sarcopenia: Inflammatory and Humoral Markers in Older Heart Failure Patients

Summary of main results.

Sarcopenia, in addition to being highly prevalent, was associated with higher levels of interleukin-6 and decreased functional capacity.

The prevalence of sarcopenia was close to that of the SICA-HF study in ambulatory patients with HF,^27^ but was higher than the results of Canteri et al. (2019).^28^ Different methods of assessing ASMM, as well as different levels of physical activity, might have affected this difference. The present study reiterates the association between sarcopenia and age in HF patients found in other studies,^29 , 28^ as well as the association between sarcopenia and lower LVEF values.^29^

It is well-known that HF can induce sarcopenia through common pathophysiological pathways that influence each other.^8^ Inflammation is a central process in HF,^30^ given that patients with HF often have low levels of chronic systemic inflammation, which can have a sustained effect on skeletal muscles.^3^ High levels of inflammatory markers, such as TNF-α, hs-CRP, and IL-6, are related to a decline in muscle mass and strength,^3 , 31^ which suggests that inflammation, which is also involved in the pathogenesis of sarcopenia, represents a essential link between these two conditions.^9^

IL-6, one of the inflammatory markers related to sarcopenia in the present study, has already been proven to be associated with muscle strength and function,^32^ in addition to having already demonstrated an association with the prognosis of this population.^30^ In chronic diseases and in older adults, IL-6 seems to be deeply implicated in the pathophysiology of declining functional capacity, which leads to the hypothesis that its deregulation could be the first step in the development of sarcopenia.^33^ Increased levels of TNF-α and IL-6 following the onset of sarcopenia in community-dwelling older adults corroborate this hypothesis.^34^ However, this is still controversial in the literature,^35 , 31^ and given the cross-sectional design of the present study, only hypotheses can be speculated about the causal role of IL-6 in sarcopenia in HF patients.

The role of inflammatory markers may also be related to the decline in anabolic hormones described in sarcopenia. In this study, lower serum IGF-1 levels were not observed in sarcopenic patients, despite the association with IL-6. One of the mechanisms by which IL-6 is linked to sarcopenia is direct interference with insulin signal transduction and inhibition of the production and biological activity of IGF-1.^36^ In the present study, none of the endocrine system markers were associated with sarcopenia, which may suggest that due to common pathologies, the specific effects of sarcopenia on anabolic hormones may not be perceived. Another possible reason may be that IGF-1 and testosterone have been considered only as continuous variables in our study, whereas the presence of a testosterone deficiency and/or low IGF-1 syndrome have not been investigated.^37^

In addition to significant differences in mean values of individual sarcopenia components between patients with and without sarcopenia, the sarcopenia group also performed more poorly on the 6-minute walk test, a well-established exercise capacity parameter with prognostic value for mortality in stable HF patients.^23^ It is reported in the literature that deficits in muscle mass, strength, and exercise capacity contribute to decreased quality of life in HF patients.^3 , 4^ In the present study, the functional capacity was associated with sarcopenia, which indicates a probable synergistic effect between the two diseases and their effect on functional capacity.

Another important issue to be considered in studies regarding sarcopenia in HF patients is the pharmacological treatment. Some standard HF medications have shown potential benefits against muscle loss. In the present study, the vast majority of individuals (above 90%) had optimized pharmacological treatment in the general analysis, which may have impacted the prevalence of sarcopenia in the sample.

Moreover, the early identification of sarcopenia in this population and the implementation of therapeutic strategies aimed at recovering muscle mass and function can contribute to a better clinical management of these individuals in order to prevent negative health outcomes.

### Limitations

One of the limitations of this study lies on the fact that patients with implantable cardioverter defibrillators/cardiac resynchronization therapy were not included because of some restrictions in the use of BIA, which, hypothetically, could have impacted the absence of greater associations with studied biomarkers. Moreover, the cross-sectional nature of the study limits conclusions about causality, although the multivariate analysis results, which were adjusted to important factors such as age, BMI, ethnicity, LVEF, and ACEI/ARB use, strengthen the idea that IL-6 could serve as a marker of sarcopenia in these patients. Another point is the limited number of patients with sarcopenia in this study. Another positive aspect of the study was the inclusion of several inflammatory and hormonal parameters, since the multifactorial nature of sarcopenia etiology in HF and the complex interaction between the two conditions most likely requires a multidimensional approach. This study also provides a precise and extensive assessment of sarcopenia in HF patients, which could help in the early detection and prevention of this condition, and guide key therapeutic approaches.

In view of the findings found in this study, the importance of including the assessment of sarcopenia in the clinical routine of this population is clear, since sarcopenia is directly related to the prognosis and progression of HF and becomes crucial for the management of these patients.

Our study aimed to evaluate sarcopenia and its association with inflammatory and humoral markers, quality of life, and functional capacity in older HF patients. Thus, we believe our results can contribute significantly toward a better understanding of this complex relationship, and can provide a preliminary basis for the prevention, diagnosis, and treatment of sarcopenia in patients with HF.

## Conclusions

In sum, this study demonstrated that sarcopenia is highly prevalent and is associated with higher levels of IL-6 and a reduction in functional capacity (according to the 6-minute walk test) in older HF patients. The results suggest that at least one of the studied inflammatory parameters may be related to a decline in strength and muscle mass in older HF patients.
